# Diagnosis and Treatment Modalities of Cholesteatomas: A Review

**DOI:** 10.7759/cureus.31153

**Published:** 2022-11-06

**Authors:** Tejas G Pachpande, Chandra Veer Singh

**Affiliations:** 1 Pathology, Jawaharlal Nehru Medical College, Datta Meghe Institute of Medical Sciences, Wardha, IND; 2 Otolaryngology - Head and Neck Surgery, Jawaharlal Nehru Medical College, Datta Meghe Institute of Medical Sciences, Wardha, IND

**Keywords:** treatment, acquired cholesteatoma, congenital cholesteatoma, pathogenesis, noncancerous cystic lesion

## Abstract

A cholesteatoma is an abnormal, noncancerous skin growth that can appear beneath the eardrum in the center of your ear. Although it might be a congenital condition, frequent middle ear infections are the main culprit. A cholesteatoma frequently appears as a cyst or sac that exfoliates the skin's outer layers. As these dead skin cells build up, the growth can enlarge and penetrate intratemporal tissues, leading to various intracranial and extracranial difficulties that can compromise facial muscles, hearing, and balance. Cholesteatomas were recognized more than three centuries ago. A cholesteatoma can develop into a serious condition if it is not treated right away. Because of its fast development and invasive nature, it can cause several consequences, some of which can be fatal for people without access to advanced medical treatment. There are no effective nonsurgical treatments available right now. It will be necessary to have a thorough awareness of both previous developments and more current ones to develop an appropriate management approach for this condition. The background information on acquired middle ear cholesteatomas is briefly reviewed in this paper. We also take into account categorization-, epidemiology-, histology-, and pathogenesis-related difficulties, and we carefully review current management and diagnosis approaches.

## Introduction and background

A cholesteatoma is a well-defined noncancerous cystic lesion that results from the aberrant development of the keratinizing squamous epithelium in the temporal bone. It is commonly characterized as *skin in the wrong place* [1]. The term *cholesteatoma* is a misnomer because it neither contains cholesterol crystals nor is a tumor to merit the suffix *oma*. A cholesteatoma results from the enzymatic activity of the cholesteatoma matrix. A cholesteatoma consists of two parts, a matrix and a central white mass. The matrix comprises keratinizing squamous epithelium resting on a tin stroma of fibrous tissue and a central white mass consisting of keratin debris produced by the matrix. Because of this, it is also known as epidermolysis or keratoma. This aberrant growth is invasive locally and has the potential to obliterate middle ear cleft structures. Additionally, squamous epithelium may become destructive in persistent infection, amplifying the osteolytic consequences of cholesteatomas [1].

In 1838, German anatomist Johannes Mueller used the term *cholesteatoma* for the first time. The word's origins translate to *cholesterol*, *fat*, and *tumor*, denoting a tumor that contains both fatty tissue and cholesterol crystals. Nomenclature is inaccurate and is regarded as the second incorrect term in otology (the first one is acoustic neuroma, given that it is, in fact, a vestibular nerve schwannoma). In addition to the fact that the tumor's nature is entirely debatable, the usage of this term is improper as cholesteatomas are derived from the keratinized squamous epithelium of the tympanic membrane and the external auditory canal, which contains no cholesterol crystals or fat [2]. A cholesteatoma is classified into two types - congenital, which is specific to childhood, and acquired, which is further subdivided into primary and secondary. In primary cholesteatomas, there is no previous history of otitis media or preexisting perforation, whereas in secondary cholesteatomas, there are preexisting perforations in pars tensa [3]. This is most commonly associated with marginal perforation or sizeable central perforation. Once a cholesteatoma invades the middle ear cleft (the middle ear cleft consists of the mastoid air cell, antrum, aditus, attic, middle ear, and eustachian tube), it invades nearby structures, which subsequently leads to enzymatic destruction of bone.

Patients may develop symptoms such as hearing loss; ear drainage, often with a bad smell; recurrent ear infections; a sensation of ear fullness; dizziness; facial muscle weakness on the side of the infected ear; and earache. A cholesteatoma is diagnosed through otoscopic examination, CT, MRI, and tympanometric and audiometric tests. Any medical treatment cannot cure it. Surgical treatment is necessary to treat this condition. Coexistent infections are treated with local and systemic antibiotics to keep the ear dry, as the surgical procedure cannot be performed when there is ear discharge. The primary goal of surgery is to remove the skin and clear the infection. Surgical methods include canal wall up (CWU) and canal wall down (CWD) mastoidectomies. This may involve reconstructing the eardrum, removing bone behind the ear, or reconstructing the hearing loss. In some cases, a second surgery may be required to ensure all cholesteatomas are terminated before the hearing bones can be rebuilt. If necessary, the second surgery will typically be performed 6 to 12 months after your first surgery.

## Review

Classification

*Congenital* *Cholesteatoma*

It develops from an embryonic epidermal cell resting in the temporal bone or the middle ear cleft. A congenital cholesteatoma can develop in the middle ear, the petrous apex, or the cerebellopontine angle, and depending on where it develops, it can cause different symptoms. Congenital cholesteatoma of the middle ear causes conductive hearing loss and manifests as a whitish mass behind an intact tympanic membrane. It can occasionally be found during a child's normal checkup or when having a myringotomy [4]. Additionally, it has the potential to spontaneously burst through the tympanic membrane and manifest as chronic suppurative otitis media with ear discharge. Congenital cholesteatomas can be seen by Levenson criteria. (The pars tensa and pars flaccida should be normal or intact, and there should be no history of perforation and no history of surgery.)

*Acquired* *Cholesteatoma*

**Figure 1:**
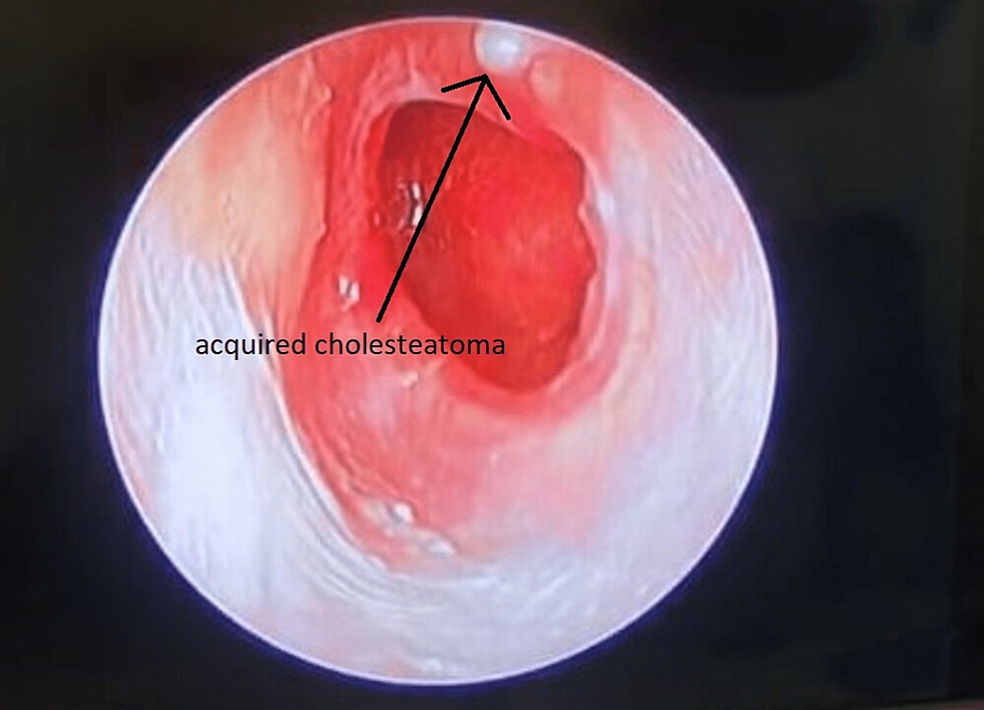
Acquired cholesteatoma. Figure credits: Tejas Pachpande

Primary acquired cholesteatoma: It is known as primary cholesteatoma because there is no prior history of otitis media or a perforation that is already present. Figure 1 shows acquired cholesteatoma. Theories on its origin include the following:

· Invagination of pars flaccida: In the attic, persistent negative pressure creates a retraction pocket where keratin debris gathers. The keratin mass enlarges toward the middle ear when infected. Thus, the proximal end of an expanding invaginated sac is an attic perforation.

· Basal cell hyperplasia: Subclinical pediatric illnesses cause the pars flaccida basal layer to proliferate. An attic perforation is then created when an expanding cholesteatoma penetrates the pars flaccida.

· Squamous metaplasia: Due to subclinical infections, the attic's normal pavement epithelium goes through metaplasia and keratinizes squamous epithelium. It has also been shown that such a shift occurs in situations of otitis media with effusion.

Secondary acquired cholesteatoma: In some instances, the pars tensa already has a hole. This frequently coexists with extensive central or posterosuperior marginal perforations. Theories on its genesis include the following:

· Migration of squamous epithelium: The external auditory canal or the tympanic membrane’s outer surface keratinizing squamous epithelium migrates through the opening into the middle ear. Tympanic annulus perforations, such as those that occur in acute necrotizing otitis media, are more prone to allow the squamous epithelium to proliferate.

· Metaplasia: Due to recurrent infections of the middle ear through the preexisting hole, the middle ear mucosa goes through metaplasia [5].

Epidemiology

The annual incidence of cholesteatomas is reported as 3 per 100,000 in children and 9.2 per 100,000 in adults with a male predominance of 1.4:1 [6]. Middle ear cholesteatomas have a higher incidence in individuals aged less than 50 years, whereas external auditory canal (EAC) cholesteatomas present predominantly at 40-70 years of age. A hereditary predisposition is probable [7]. There is a high prevalence among white individuals, and cholesteatomas are rarely detected in the Asian, American Indian, and Alaskan Eskimo populations [8].

Histopathology

At the macroscopic level, a cholesteatoma presents as a whitish ovoid or round friable mass with a thin wall that contains pultaceous or a macerated substance. The lesion is divided into three layers at the microscopic level: the cystic content, the matrix, and the perimatrix [9]. The cyst's contents, which include completely differentiated anucleate keratin squames mixed with sebum material as well as purulent and/or necrotic debris, are the main part of cholesteatomas. Squamous epithelium that is stratified and hyperproliferative makes up the cholesteatoma matrix. The cholesteatoma epithelium is made up of a granular layer, a lucid layer, a spinal layer, and a basal layer, similar to the skin. The perimatrix (lamina propria), the epithelial connective tissue's outermost layer, is composed of fibrocytes, lymphocytes, histiocytes, plasma cells, and neutrophil leucocytes, among other inflammatory cells [10].

Pathogenesis

Explanations for the pathogenesis of a cholesteatoma can be categorized into the following four groups: (1) the invagination theory (the retraction pocket theory), (2) the theory of epithelial invasion or migration (the immigration theory), (3) the theory of squamous metaplasia, and (4) the theory of basal cell hyperplasia (the papillary ingrowth theory) [11]. According to the invagination theory, pars flaccida's retraction pockets, which are brought on by the middle ear negative pressure and possibly repeated inflammation, are the predecessors of cholesteatoma [12]. A cholesteatoma develops as a result of the following buildup of desquamated keratin in a growing retraction pocket. The immigration argument disputes the invagination idea by positing that eardrum perforations precede cholesteatomas. A cholesteatoma develops when the squamous epithelium of the eardrum invades or migrates into the middle ear through traumatic or intentional damage to the tympanic membrane [8]. The middle ear mucosa, on the other hand, can metaplastically transform into keratinizing epithelium, which ultimately leads to the formation of cholesteatomas, according to the squamous metaplasia theory [13]. Tympanic membrane lysis and perforation caused by an enlarged cholesteatoma may eventually result in a situation that resembles an acquired cholesteatoma. According to the basal cell hyperplasia theory, cholesteatomas form when keratin-filled microcysts, buds, or pseudopods that arise in the pars flaccida epithelium's basal layer infiltrate the subepithelial tissue of Prussak's gap [14].

Diagnosis

The following tests are carried out to check for hearing loss: An audiogram is carried out to assess the eardrum, middle ear, and hearing. CT scan of the ear helps to see if there is damage to the ear bones. MRI is done if there is a concern that the cholesteatoma is spreading through the skull base.

*Otoscopic* *Examination*

Otoscopy, which includes video-otoscopy, otomicroscopy, and video-telescopy, is the most direct and effective technique for inspecting the eardrum. Making certain there is sufficient brightness and light during an otoscopic examination is essential [15]. The ear canal must be completely cleared of all materials, such as cerumen, crust, debris, and granulation tissue, to inspect concealed regions and prevent the wrong diagnosis of cholesteatoma. The whole eardrum and the attic and posterosuperior quadrant, which are the areas most usually recognized as the cause of acquired cholesteatoma, must be carefully examined (stretching to its outside margins) [16]. It is often present with an accumulation of squamous debris in the pocket. Sometimes granulation tissue may appear from the mucosa adjacent to the cholesteatoma, a granular polyp within an ear canal symbolizes a cholesteatoma until it is proven. Precautions must be taken while removing the polyp from the ear as the cholesteatoma may be attached to various important structures such as the ossicle or facial nerve. This examination's goal is to find any lesions that might be cholesteatomatous or their predecessor, a retraction pocket. Clinicians need to be aware of lesions linked to cholesteatomas, and otitis external may show similar lesions on otoscopic images during outpatient evaluation [17].

Radiological Examination

CT: The main imaging technique used in this field to assess the severity of the condition and aid in the development of a surgical strategy is CT. Repeated HRCT scans should be performed before surgery to determine the severity of the illness, any potential osseous damage, any malformations of the body (e.g., middle ear hypoplasia, jugular bulb variations, bony dehiscence of the facial nerve and anomalies of its natural course, sclerotic or diploic mastoids, anterior sigmoid sinuses, and low-lying segments) [18], and other complications, such as tegmen dehiscence and labyrinthine fistulas [19]. CT scans are the preferred way for determining if there is bone involvement; however, this technology has certain limitations when it comes to assessing soft tissue alterations, such as those brought on by membranous labyrinthine or intracranial involvement [20]. Additionally, the frequent opacification seen in CT scans after tympanomastoid surgery might reduce the accuracy of evaluations of postoperative recurrence or residual illness [21]. However, we cannot distinctly distinguish between granulation tissue and cholesteatoma in this examination.

MRI: Due to the various pulse sequences used in MRI, complementing information is provided, improving tissue distinction [22]. Cholesteatomas often exhibit hypointense/isointense on T1WI and hyperintense on T2WI MR imaging signal-intensity characteristics when compared to brain tissue. On T2WI, bloody serous or proteinaceous fluid as well as granulation or scar tissue in an ear that has already undergone surgery exhibits hyperintense signal intensity [23]. When compared to the surrounding granulation tissue, cholesteatomas occasionally show less signal strength on T2WI or constructive interference in fast imaging employing steady-state acquisition (FIESTA); however, they may also be undetectable on these sequences. It could be able to differentiate on contrast-enhanced T1WI, [24] because cholesteatomas do not exhibit contrast enhancement but granulation tissues do. Standard MRI sequences, however, might not be able to detect cholesteatomas in a temporal bone that has recently undergone surgery [25].

*Ancillary* *Diagnostic* *Tools*

Tympanometric and audiometric tests are ancillary diagnostic methods that can be used to assess cholesteatomas. Conducted hearing deficiencies are discovered via audiometric testing. Labyrinthitis may be linked to the symptom of mixed hearing loss with a sensorineural component [26]. Sensorineural hearing loss can be ruled out by the Rinne test, while conductive hearing loss can be detected by the Weber test. The hearing may be normal if there is no involvement of the ossicular chain. The cholesteatoma can transmit sound and fill the ossicular gap, making hearing look unaffected or just slightly diminished even when the ossicles are damaged. This unusual phenomenon is referred to as *silent cholesteatoma*, *conductive cholesteatoma*, or *cholesteatoma hearer* [27]. To assess the health of the middle ear, tympanometric testing is frequently combined with audiometric testing. Tympanometric data may indicate a perforated eardrum, which is less common in children than adults, and decreased compliance on the afflicted side. Similar to audiometric testing, tympanometric testing may not always accurately reflect the state of the middle ear. Unfortunately, cholesteatomas cannot be definitively diagnosed using a specific tympanometric change pattern [28].

Treatment

CWU Procedure 

The posterior bone meatal wall is left intact while the illness is removed using a combined approach through the meatus and mastoid, preventing an exposed mastoid cavity [29]. It causes dry ears and enables simple regeneration of the hearing system. However, there is a chance that some cholesteatomas will remain. Long-term follow-up is crucial as there is a very high risk of residual or recurrent cholesteatoma. After around six months, some surgeons even suggest routine re-exploration. Only under a few circumstances, CWU methods are suggested [29]. The disease is permanently eradicated as part of the combined approach or intact canal wall mastoidectomy, as well as through cortical mastoidectomy and the posterior tympanotomy approach, which entails making a window between the mastoid and the middle ear through the sinus tympani in the facial recess [30]. 

CWD Procedure

So that the affected region is completely exteriorized, they leave the external auditory canal and the mastoid cavity uncovered. Atticotomy, modified radical mastoidectomy (MRM), and radical mastoidectomy are the procedures most frequently used to treat atticoantral illness [31]. One should not consider going in a wet condition such as swimming, bathing, or rain after mastoid surgery for cholesteatoma as it may lead to various complications such as otorrhea (ear drainage), otalgia (ear pain), vertigo (the sensation that you or the environment around you is moving or spinning), and dizziness (feeling faint, woozy, weak, or unsteady) [32].

Reconstructive Surgery and Conservative Treatment

Myringoplasty and tympanoplasty can help restore hearing. It may be carried out as part of the initial surgery or as a subsequent operation [33]. It can be used in some circumstances, when the cholesteatoma is tiny and easily accessible for suction clearing under the operating microscope, despite having a limited function in the therapy of cholesteatoma [34]. Regular checks and repeated suction clearing are crucial. Additionally, it can be tested on patients over the age of 65 years, those who cannot undergo general anesthesia, and those who refuse surgery [35]. Additionally, polyps and granulations can be chemically cauterized with substances like silver nitrate or trichloroacetic acid, or they can be surgically removed with cup forceps. Aural toilet and dry ear precautions, among others, are also crucial [36].

## Conclusions

To facilitate treatment and management, careful and early evaluation is the key to an early diagnosis. Both classic and current evaluation methods such as audiometric tests, tympanometric tests, X-rays, CT scans, and MRIs can be used. For cholesteatoma to be discovered early, greater awareness is required. Symptoms such as a persistent or recurring watery, often smelly, discharge from the ear, which may be irregular, and gradual loss of hearing in the affected ear may be seen. A thorough understanding of the crucial anatomical structures, their relationships with the middle ear function and management, the histology involved, and the specific surgical procedure is required. CWU and CWD procedures that may need to be customized for each patient, depending on the severity of the disease involved, are all necessary for this process to go smoothly and preserve hearing.

## References

[1] Dornelles C, Costa SS, Meurer L, Schweiger C (2005). Some considerations about acquired adult and pediatric cholesteatomas. Braz J Otorhinolaryngol.

[2] Sudhoff H, Dazert S, Gonzales AM (2000). Angiogenesis and angiogenic growth factors in middle ear cholesteatoma. Am J Otol.

[3] Sie KC (1996). Cholesteatoma in children. Pediatr Clin North Am.

[4] Nevoux J, Lenoir M, Roger G, Denoyelle F, Ducou Le Pointe H, Garabédian EN (2010). Childhood cholesteatoma. Eur Ann Otorhinolaryngol Head Neck Dis.

[5] Tos M (1988). Incidence, etiology and pathogenesis of cholesteatoma in children. Adv Otorhinolaryngol.

[6] Nelson M, Roger G, Koltai PJ (2002). Congenital cholesteatoma: classification, management, and outcome. Arch Otolaryngol Head Neck Surg.

[7] Potsic WP, Korman SB, Samadi DS, Wetmore RF (2002). Congenital cholesteatoma: 20 years' experience at The Children's Hospital of Philadelphia. Otolaryngol Head Neck Surg.

[8] Kemppainen HO, Puhakka HJ, Laippala PJ, Sipilä MM, Manninen MP, Karma PH (1999). Epidemiology and aetiology of middle ear cholesteatoma. Acta Otolaryngol.

[9] Ferlito A (1993). A review of the definition, terminology and pathology of aural cholesteatoma. J Laryngol Otol.

[10] Lim DJ, Saunders WH (1972). Acquired cholesteatoma: light and electron microscopic observations. Ann Otol Rhinol Laryngol.

[11] Sudhoff H, Tos M (2000). Pathogenesis of attic cholesteatoma: clinical and immunohistochemical support for combination of retraction theory and proliferation theory. Am J Otol.

[12] Karmody CS, Northrop C (2012). The pathogenesis of acquired cholesteatoma of the human middle ear: support for the migration hypothesis. Otol Neurotol.

[13] Chole RA, Tinling SP (1985). Basal lamina breaks in the histogenesis of cholesteatoma. Laryngoscope.

[14] Yamamoto-Fukuda T, Takahashi H, Koji T (2011). Animal models of middle ear cholesteatoma. J Biomed Biotechnol.

[15] Chang P, Kim S (2008). Cholesteatoma --- diagnosing the unsafe ear. Aust Fam Physician.

[16] Hassman-Poznańska E, Kurzyna A, Trzpis K, Poznańska M (2012). The status of the contralateral ear in children with acquired cholesteatoma. Acta Otolaryngol.

[17] Dannatt P, Jassar P (2013). Management of patients presenting with otorrhoea: diagnostic and treatment factors. Br J Gen Pract.

[18] Magliulo G, Colicchio MG, Appiani MC (2011). Facial nerve dehiscence and cholesteatoma. Ann Otol Rhinol Laryngol.

[19] Manolis EN, Filippou DK, Tsoumakas C (2009). Radiologic evaluation of the ear anatomy in pediatric cholesteatoma. J Craniofac Surg.

[20] Sone M, Yoshida T, Naganawa S (2012). Comparison of computed tomography and magnetic resonance imaging for evaluation of cholesteatoma with labyrinthine fistulae. Laryngoscope.

[21] Jindal M, Riskalla A, Jiang D, Connor S, O'Connor AF (2011). A systematic review of diffusion-weighted magnetic resonance imaging in the assessment of postoperative cholesteatoma. Otol Neurotol.

[22] Corrales CE, Blevins NH (2013). Imaging for evaluation of cholesteatoma: current concepts and future directions. Curr Opin Otolaryngol Head Neck Surg.

[23] Venail F, Bonafe A, Poirrier V, Mondain M, Uziel A (2008). Comparison of echo-planar diffusion-weighted imaging and delayed postcontrast T1-weighted MR imaging for the detection of residual cholesteatoma. AJNR Am J Neuroradiol.

[24] Martin N, Sterkers O, Nahum H (1990). Chronic inflammatory disease of the middle ear cavities: Gd-DTPA-enhanced MR imaging. Radiology.

[25] Kimitsuki T, Suda Y, Kawano H, Tono T, Komune S (2001). Correlation between MRI findings and second-Look operation in cholesteatoma surgery. ORL J Otorhinolaryngol Relat Spec.

[26] Schwartz RH, Grundfast KM, Feldman B, Linde RE, Hermansen KL (1984). Cholesteatoma medial to an intact tympanic membrane in 34 young children. Pediatrics.

[27] Albera R, Canale A, Piumetto E (2012). Ossicular chain lesions in cholesteatoma. Acta Otorhinolaryngol Ital.

[28] Albera R, Canale A, Piumetto E, Lacilla M, Dagna F (2012). Ossicular chain lesions in cholesteatoma. Acta Otorhinolaryngol Ital.

[29] Dornhoffer JL, Friedman AB, Gluth MB (2013). Management of acquired cholesteatoma in the pediatric population. Curr Opin Otolaryngol Head Neck Surg.

[30] Tomlin J, Chang D, McCutcheon B, Harris J (2013). Surgical technique and recurrence in cholesteatoma: a meta-analysis. Audiol Neurootol.

[31] Carlson ML, Latuska RF, Pelosi S (2014). Evolving considerations in the surgical management of cholesteatoma in the only hearing ear. Otol Neurotol.

[32] Fyrmpas G, Tsetsos N, Poutoglidis A, Alghoj A, Vlachtsis K (2022). What is the impact of mastoid surgery on swimming?. Clin Otolaryngol.

[33] Roger G, Denoyelle F, Chauvin P, Schlegel-Stuhl N, Garabedian EN (1997). Predictive risk factors of residual cholesteatoma in children: a study of 256 cases. Am J Otol.

[34] Tarabichi M, Nogueira JF, Marchioni D, Presutti L, Pothier DD, Ayache S (2013). Transcanal endoscopic management of cholesteatoma. Otolaryngol Clin North Am.

[35] Quaranta N, Fernandez-Vega Feijoo S, Piazza F, Zini C (2001). Closed tympanoplasty in cholesteatoma surgery: long-term (10 years) hearing results using cartilage ossiculoplasty. Eur Arch Otorhinolaryngol.

[36] Hagr A (2007). BAHA: bone-anchored hearing aid. Int J Health Sci (Qassim).

